# Clinics, Epidemiology and Genetics of Retinitis Pigmentosa

**DOI:** 10.2174/138920211795860080

**Published:** 2011-06

**Authors:** Francesco Parmeggiani

Inherited retinal dystrophies represent the most frequent hereditary disorders of the ocular posterior segment. The chronic eye diseases included in this heterogeneous group of genetic degenerative disorders of the retina are also habitually named retinitis pigmentosa, a term coined by Donders more than 150 years ago.

Taking into account all the different forms of retinitis pigmentosa (RP), among the general population, their total prevalence is variably reported in one case for each 2500-7000 persons. In view of that, RP should be labeled as rare or orphan disease even if, for several reasons, it could be considered very atypical within this category of pathologies; firstly given that a world-wide shared consensus on the definition of rare disease does not exist, but also because RP represents one of the most common causes of blindness or severe low-vision in people from 20 to 60 years old. In addition, it is not true that RP is less known or studied than other more frequent eye disorders, and the nearly 7000 references, which appear using “retinitis pigmentosa” in a PubMed search, demonstrate that RP is characterized by a high “desirability” for clinical and experimental researches. On the other hand, it is true that many clinical and welfare problems are common to all RP forms and, above all, that RP requires specific and continuative cares, the fulfillment of which is impossible without a considerable public support participation. Moreover, despite the outstanding scientific advances achieved in the knowledge of RP during the last three decades, evidence-based therapies do not exist for this terrible retinal neuro-degeneration. In other words, RPs are not sufficiently rare as to result in a low research interest, but they are so genotypically intricate and/or phenotypically severe that become very hard to face. At present, the numerous forms of RP are considered the most complex category of retinal diseases. They can be transmitted by all types of monogenic inheritance (autosomal dominant, autosomal recessive, X-linked), even if many cases are diagnosed in patients with no report of affected relatives. These genetic degenerations of the retina are sometimes associated with various non-ocular disorders (syndromic RP), and are characterized by: i) remarkable taxonomic heterogeneity; ii) frequent phenotypic inter- or intra-familial variability; iii) large genotypic multiplicity which becomes more evident examining different ethnic clusters. In fact, although more than 200 causative mutations of RP have been hitherto discovered in more than 100 different genes, the molecular defect is identifiable in just about the 50% of the affected patients.

The comprehensive appraisal of these latter issues leads to a high difficulty in planning adequate clinical researches and, unfortunately, to the development of an indecorous market of vain therapeutic attempts instigating false expectations in many RP patients. An attenuation of the aforementioned trouble could rise from two main ways of acting: i) when a factual therapeutic advancement is established in animal models of RP, immediate consideration should be also given to possibly realize, within a reasonable timeline, a clinical trial based on that experimental evidence; ii) the prescriptive attitude toward this rare disease should be comprehensively based on scientific, deontological, rehabilitative and psychological aspects, also because the application of either evidence-based medicine or complementary and alternative medicine criteria may be not appropriate in the context of RP decision-making. In this manner also, even if just partly, avoid that the patient with RP must be suffering a double injury: being affected by a severe disease transmissible to own children, and being inadequately managed from a clinical point of view.

Despite the good purposes to allocate considerable resources for both research and welfare of rare diseases, in several developed countries, as well as in the majority of developing countries, the denomination of RP with the adjective “rare” should be replaced by the adjective “orphan”, which deplorably means that it is still absent a broad diffuse socio-sanitary network dedicated to patients with RP and their families. The concrete carrying out of the aforementioned purposes will be not practicable without a coordinated collaboration between specialized ophthalmologists, geneticists and epidemiologists, necessary to exactly evaluate the burden of RP on both general population and Health System, and to finally facilitate the whole management of this vision-threatening disease.

The clinico-genetic investigations on a complex and insidious disease, such as RP, might be more effective by planning multicenter research projects, in which the presence of multidisciplinary work-teams becomes a very important aspect. In fact, a large sample size alone is not a guarantee for a reliable genetic association study, and the genetic epidemiology of RP still has many challenges to face. Among these, issues such as the standardization of clinical phenotyping, the novel genotyping arrays, the second-generation sequencing techniques, the epidemiologic puzzles and the preparation of targeted studies of screening should be continuously focused, updated and shared.

Phenotyping of both non-syndromic and syndromic forms of RP may be related to several clinical difficulties and/or organizational problems, which should be preferentially encountered in specialized referral Centers for RP. The existence of these landmarks dedicated to the RP patients, to their families and to the associations of patients with RP, can represent a pivotal key-point to complete as possible the biobanking inside each Health catchment’s area. In RP referral Center, the appropriateness of routine clinical practice is of critical importance to maximize: i) the reliability of both epidemiological and bio-bank registers (involving the proband and, if possible or applicable, other members of his/her genealogic tree); ii) the chance of success and the cost/benefit ratio of the multifaceted DNA analyses. National and international scientific networks should be structured and/or enlarged to efficiently face the new diagnostic and therapeutic challenges for patients suffering from RP.

